# Coronary Microvascular Dysfunction in Diabetes Mellitus: Pathogenetic Mechanisms and Potential Therapeutic Options

**DOI:** 10.3390/biomedicines10092274

**Published:** 2022-09-14

**Authors:** Teresa Salvatore, Raffaele Galiero, Alfredo Caturano, Erica Vetrano, Giuseppe Loffredo, Luca Rinaldi, Christian Catalini, Klodian Gjeloshi, Gaetana Albanese, Anna Di Martino, Giovanni Docimo, Celestino Sardu, Raffaele Marfella, Ferdinando Carlo Sasso

**Affiliations:** 1 Department of Precision Medicine, University of Campania Luigi Vanvitelli, Via De Crecchio 7, I-80138 Naples, Italy; 2 Department of Advanced Medical and Surgical Sciences, University of Campania Luigi Vanvitelli, Piazza Luigi Miraglia 2, I-80138 Naples, Italy

**Keywords:** diabetes mellitus, microvascular complication, endothelial dysfunction, glucose lowering drugs

## Abstract

Diabetic patients are frequently affected by coronary microvascular dysfunction (CMD), a condition consisting of a combination of altered vasomotion and long-term structural change to coronary arterioles leading to impaired regulation of blood flow in response to changing cardiomyocyte oxygen requirements. The pathogenesis of this microvascular complication is complex and not completely known, involving several alterations among which hyperglycemia and insulin resistance play particularly central roles leading to oxidative stress, inflammatory activation and altered barrier function of endothelium. CMD significantly contributes to cardiac events such as angina or infarction without obstructive coronary artery disease, as well as heart failure, especially the phenotype associated with preserved ejection fraction, which greatly impact cardiovascular (CV) prognosis. To date, no treatments specifically target this vascular damage, but recent experimental studies and some clinical investigations have produced data in favor of potential beneficial effects on coronary micro vessels caused by two classes of glucose-lowering drugs: glucagon-like peptide 1 (GLP-1)-based therapy and inhibitors of sodium-glucose cotransporter-2 (SGLT2). The purpose of this review is to describe pathophysiological mechanisms, clinical manifestations of CMD with particular reference to diabetes, and to summarize the protective effects of antidiabetic drugs on the myocardial microvascular compartment.

## 1. Introduction

Microvascular dysfunction is a hallmark of diabetes mellitus (DM) closely related to microangiopathy, a chronic complication that affects 25 times more diabetic patients than not diabetics, impairing their quality and duration of life [1].

Despite the different pathogenesis of type 1 (T1DM), type 2 (T2DM), and other types of diabetes, all states of hyperglycemia share the dysfunction of microvessels as a common chronic feature due to the pronounced sensitivity of this vascular compartment to oxidative stress and the inflammatory response to high circulating glucose levels. Accordingly, two milestones of clinical research in diabetes, the Diabetes Control and Complications Trial in T1DM and the United Kingdom Prospective Diabetes Study in T2DM, underscored the role of improved glycemic control for decreasing the incidence and progression of microvascular complications in diabetes [2,3].

Microangiopathy is more precocious and frequent in the course of T2DM. This has been clearly demonstrated in adolescents with durations of T2DM of just over a year, who had a significantly greater prevalence of microalbuminuria than adolescents with durations of T1DM of about seven years, despite comparable or better glycemic control in T2DM subjects [4,5]. Adolescents with T2DM had greater prevalence of obesity, hypertension, and dyslipidemia, and cross-sectional studies of adult populations demonstrated that these conditions were associated with increased prevalence of retinopathy in non-diabetic people [4,6]. Thus, it can be inferred that, in addition to hyperglycemia, other components of cardiometabolic disease may increase the risk of development of diabetic microvasculopathy. On the other hand, the Joslin Diabetes Center 50-Year Medalist Study showed that 30% to 35% of patients with a > 50-year history of T1DM did not develop significant microvascular injury, regardless of hemoglobin A1c (HbA1c) levels and other classical risk factors for diabetic vasculopathy [7]. Presumably, unidentified genetic or other endogenous processes that lead to microvascular damage may play a part [8].

The systemic microvasculature establishes a paracrine, regulatory, and reciprocal relationship with perivascular tissues to form microvascular units which are disrupted early in the course of diabetes and metabolic syndrome. Thereby, tissues become dysfunctional because of both damaged perfusion and tissue-specific responses to microvascular injury orchestrated by many systemic and local signaling processes [9].

The organ-specific manifestations of microvasculopathy depend on the blood perfusion needs, importance of impaired functions, and entity of protective responses implemented at the tissue level. Repair processes are activated through the enhancement of antioxidant defenses to counteract injury caused by reactive oxygen species (ROS), and recruitment of progenitor cells to replace those irreversibly damaged [9]. These protective mechanisms may explain why metabolic and inflammatory alterations induce early dysfunction but yet require more than a decade to yield anatomic changes to the microvasculature.

Though diabetic microangiopathy classically identifies with retinopathy, nephropathy, and neuropathy, in every diabetic patient, hyperglycemia simultaneously affects the body’s microvascular compartments to a greater or lesser degree [10,11,12,13,14]. This is true to such an extent, that if eye, kidney, or peripheral nerves appear to be clinically damaged, it is safe to assume that other tissues are involved, even if only at a preclinical stage [15].

The microvascular bed provides a much larger endothelial surface area per gram of myocardium than other tissues, and coronary microvascular endothelial cells (CMECs) account for approximately 1/3 of the total heart cells. This explains the great importance of the microcirculatory compartment to the process of the cardiac muscle delivering oxygen, nutrients, and hormones, and to the removal of metabolic end products [16]. Patients with T1DM and T2DM have a high prevalence of CMD, a condition covering a wide spectrum of clinical situations, featuring an altered vasomotion of the coronary arterioles that involves endothelium- and smooth muscle-mediated mechanisms and long-term structural changes, leading to impaired vasodilatory capacity and/or enhanced vasoconstriction. As a consequence, the regulation of blood flow in response to changing cardiomyocyte oxygen requirements from rest to stress is altered, and blood supply inadequate.

Importantly, DM patients with CMD have a poorer prognosis, with a higher rate of hospitalization for heart failure (HF) and a greater risk of sudden cardiac death and myocardial infarction than those without, even in the absence of obstructive coronary artery disease (CAD) [17,18]. It has been observed that diabetic patients with CMD show mortality rates at least as high as those of non-diabetic patients with known obstructive CAD [19]. Moreover, people with diabetes or metabolic syndrome, when stratified by the severity of metabolic impairment, have a stepwise increase in CMD and risk for coronary events [20]. Myocardial flow reserve (MFR), a significant marker of coronary microvascular dysfunction, emerged as a predictor of adverse CV events and HF with preserved ejection fraction (HFpEF) [21,22].

This review deals with the diabetic microvasculopathy of cardiac muscle by outlining the pathogenic mechanisms and structural/functional changes, as well as clinical manifestations and assessment. Considering the lack of effective treatments for this complication, the positive effects of some current glucose lowering drugs on the coronary microvessel compartment are described.

## 2. Pathophysiology of CMD in Diabetes

**Figure 1:**
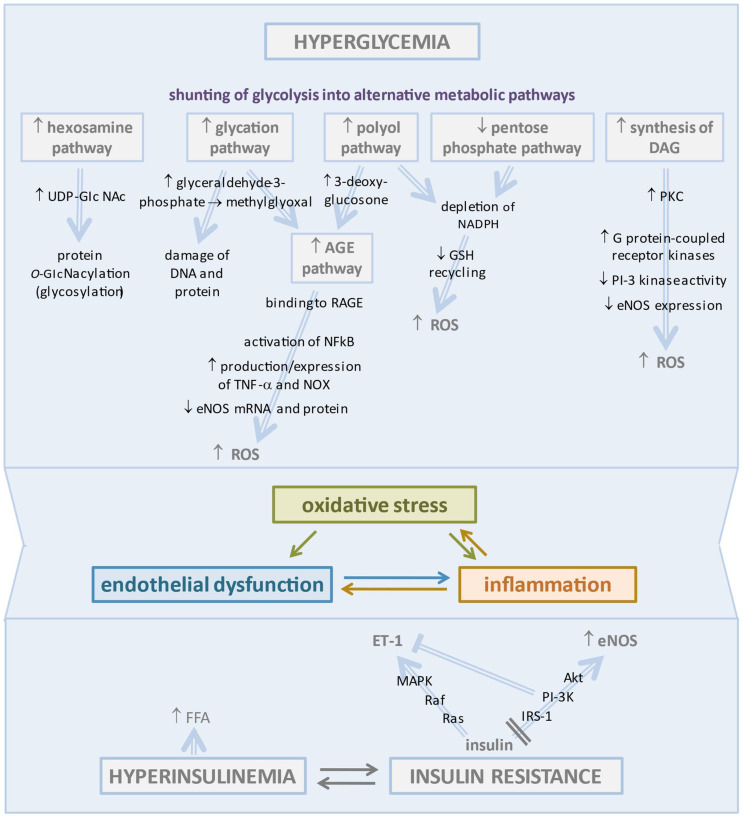
At the top: changes induced in the vascular endothelium by hyperglycemia. Under hyperglycemic conditions, the excess of glucose is shunted in a series of alternative metabolic pathways that are associated with harmful effects, mainly the generation of large amounts of ROS leading to oxidative stress and triggering a worsening vicious cycle of inflammation and endothelial dysfunction. At the bottom: Insulin resistance, the core defect of type 2 diabetes, contributes to endothelial dysfunction by reducing eNOS activity and removing the brake on ET-1 production. The arrows indicate the sequence of pathophysiological events that lead to the alterations described above.

Endothelial dysfunction associated with diabetes is the most likely primary originating cause of CMD. This process was long ago indicated by the impaired coronary micro-vascular response to intracoronary acetylcholine infusion and to sympathetic stimulation by cold pressor test in diabetic subjects without significant CAD and was clearly documented as an early defect in animal models of T2DM or metabolic syndrome [23,24,25]. A series of multifaceted mechanisms may disrupt the physiologic regulation of the endothelial function and vasomotor tone of coronary microcirculation in diabetes, primarily chronic hyperglycemia/glucotoxicity, and impaired insulin signaling, two factors that increase oxidative stress and create a proinflammatory substrate promoting CMD [26] (Figure 1).

### 2.1. Hyperglycemia-Induced Changes in Vascular Endothelium

ECs are more susceptible to hyperglycemia-induced damage than other cell types, since glucose enters them through the GLUT-1 transporter independently from insulin, largely independent of its extracellular concentrations [27,28].

Hyperglycemia is a key mediator of CMD in diabetes, as indicated by the significant correlation of MFR with average fasting glucose and HbA1c in diabetic people [29]. Likewise, observations of human atrial samples showed impairment of the baseline endothelium-dependent dilation of coronary arterioles in patients with uncontrolled diabetes compared to those with controlled diabetes or no diabetes [30].

Exposure of ECs to high glucose (HG) reduces the mRNA and protein levels of GLUT1, promotes glycolysis into lactate rather than pyruvate, and downregulates hexokinase, a rate-limiting enzyme in glycolysis leading to reduced ATP production and cytoplasmic/mitochondrial calcium overload [31,32].

In particular, HG concentrations in diabetic ECs promote the shunting of glycolytic flux into alternative metabolic pathways leading to accumulation of ROS beyond the cell’s ability to overcome oxidative stress.

Activation of the polyol pathway determines the metabolic diversion of high intra-cellular glucose to sorbitol by aldose reductase. This enzyme uses NADPH as a co-factor depleting the cellular stores required for glutathione reductase in order to generate reduced glutathione (GSH), a critical cellular antioxidant compound [33].

Importantly, the polyol pathway supplies substrates for the synthesis of advanced glycation end products (AGEs) [34]. However, the major precursor of AGEs is methylglyoxal, a highly reactive α-dicarbonyl compound derived from glyceraldehyde-3-phosphate and responsible for protein and DNA damage. Excess AGEs in diabetes bind to the receptor for AGE (RAGE) expressed on inflammatory T cells, leading to activation of NFκB and stimulation of inflammatory responses and apoptotic pathways in ECs [35]. These detrimental effects were well demonstrated in a T2DM murine model that presented increased expression of RAGE and decreased endothelium-dependent vasodilation to acetylcholine (Ach), an effect partially reversed by administration of soluble RAGEs, to which AGE were bound rather than to tissutal RAGEs [36]. The study also demonstrated that AGE/RAGE signaling increased the production/expression of TNF-α and oxidative stress markers, including NADPH oxidase (NOX) [36]. In an in vitro study, the treatment of human coronary artery endothelial cells (HCAECs) with AGEs resulted in the reduction of eNOS mRNA and protein and of NO production, as well as resulting in increased NOX activity and O^−2^ levels with less activity of catalase and superoxide dismutase (SOD) [37].

Increased hexosamine pathway flux produces the activated amino sugar UDP-GlcNAc that is a critical substrate for protein O-GlcNA cylation. This is a dynamic and reversible post-translational glycosylation of serine and threonine residues in target proteins that control angiogenesis and a range of cellular processes, including inflammation [38,39].

Pentose phosphate is a glycolytic side pathway with a nonoxidative phase producing pentoses and an oxidative phase supplying NADPH for GSH recycling. In hyperglycemia, the glucose-6-phosphate dehydrogenase-dependent entry of glucose into this pathway is impaired, leading to exacerbation of oxidative stress and endothelial dysfunction [40].

Through the raised triose phosphate concentrations generated by glycolysis, hyperglycemia stimulates de novo synthesis of the lipid second messenger diacylglycerol (DAG) responsible for the hyperactivation of several protein kinase C (PKC) isoforms (β, δ, and θ), which contributes to coronary endothelial dysfunction in diabetes [41]. The coronary vessels of T1DM rats exhibited elevated protein expression of PKC and enhanced vasoconstriction by endothelin mitigated by PKC inhibition [42]. The activation of PKC in the vascular tissues, as occurs in diabetes and insulin resistance, may inhibit PI-3 kinase activity and eNOS expression, likely through the activation of G protein-coupled receptor kinases, which negatively regulate the insulin-mediated Akt/eNos pathway and contribute to oxidative stress [43,44]. These effects were documented in porcine coronary microvessels, wherein the activation of PKC impaired NO-mediated vasodilation via the production of superoxide anion (O^−2^) from xantine oxidase (XO) and JNK signaling through Rho kinase activation [45]. The hyperglycemia-mediated activation of the DAG-PKC signaling pathway seems to be implicated in the promotion of endothelial barrier dysfunction [46]. In a study on human umbilical vein endothelial cells (HUVECs), hyperglycemia determined phosphorylation of the myosin light chain by PKC, which in turn caused disruption of adherens junctions [47]. The increase in PKC activity as well as methylglyoxal concentrations promoted the activation of the NFkB innate inflammatory pathway, which drove the injury process forward [48].

### 2.2. Insulin Resistance in the Pathogenesis of CMD

CMD may precedes hyperglycemia in the development of T2DM, since factors other than glucose, namely insulin resistance and hyperinsulinemia in prediabetes, may impair microvascular endothelium-dependent coronary and skeletal vasodilatation. In turn, microvascular disease contributes to the progression of prediabetes to T2DM by reducing the delivery of insulin and glucose to skeletal muscles [49].

Under normal conditions, insulin binding to the insulin receptor of Ecs activates two pathways, IRS-1/PI3K/Akt signaling, which mediates vasodilator actions through stimulation of eNOS, and the MAPK signaling pathway which promotes endothelin-1 secretion [50]. Insulin resistance does not affect the MAPK pathway, but it selectively impairs PI3K-dependent insulin signaling, which contributes to the positive associations between insulin resistance and endothelial dysfunction [51]. Actually, insulin resistance favors both ED and hepatic steatosis, which in turn increases insulin resistance, resulting in a vicious circle [52,53]. This linkage has been confirmed by recent studies which have shown that the reduction of hepatic insulin resistance induced by antiviral drugs results in a reduction in the incidence of type 2 diabetes, MACE, and hepatocellular carcinoma [54,55,56,57]. These important observations confirm the close pathophysiological correlation between insulin resistance and ED.

A series of findings support the involvement of coronary microvessels in insulin resistance. In healthy humans, insulin increased coronary flow reserve by ~20–26%, an effect which was maintained in young T1DM patients without microvascular complications or autonomic neuropathy but blunted in patients with obesity or T2DM and in acute insulin-resistant states induced by lipid infusion [58,59,60,61,62,63,64]. In a rat model of metabolic syndrome, coronary small arteries exhibited endothelial dysfunction associated with decreased eNOS expression and NO production compared to lean controls [65]. In a porcine model of chronic myocardial ischemia, coronary arterioles from diabetic pigs had impaired endothelium-dependent but intact endothelium-independent vasodilator responses [66].

### 2.3. Decreased NO Bioavailability in Diabetes

The hallmark of diabetic endothelial dysfunction is the loss of NO-dependent vasodilatory response due to reduced activity or expression of eNOS and, to a greater extent, to scavenging inactivation of NO by excess free radicals [67,68].

Several mechanisms may explain how NO bioavailability decreases in diabetic Ecs.

First, quenching of NO by AGEs has long been reported in experimental diabetes [69]. Moreover, hyperglycemia activates NOX, an enzyme which catalyzes the reduction of oxygen to O^−2^ using NADPH as an electron donor and is involved in the hyperglycemia-mediated increase of cytoplasma-derived ROS [70,71,72]. Excess O^−2^ can rapidly react with NO to form peroxynitrite (ONOO−) with further reduction of NO bioavailability or may be converted to hydrogen peroxide (H_2_O_2_) allowing for the generation of highly reactive hydroxyl radicals. These last, together with peroxynitrite, amplify oxidative injury, damaging proteins, lipids, DNA, and, especially, prostacyclin synthase and eNOS [26]. In particular, the oxidation of the zinc-thiolate center of eNOS results in eNOS uncoupling, a process wherein eNOS produces O^−2^ instead of NO in endothelial cells [73]. Moreover, peroxynitrite leads to the oxidation of BH4 to BH3 radical, which further diminishes eNOS activity [74]. Asymmetric dimethylarginine, an endogenous eNOS inhibitor, is found at elevated levels in T2DM patients due to the impairment of dimethylarginine dimethylaminohydrolase action by hyperglycemia. In addition, diabetes is associated with increased activity of arginase, an enzyme expressed in the blood vessels where it competes with NOS for its substrate, L-arginine [75,76,77].

Along with the NO deficit, diabetes is associated with reduced prostacyclin secretion and an increase of contracting factors, including prostanoids, ET-1, thromboxane A2, and PGF2α [78]. These products, in turn, upregulate NOX and phosphodiesterases of type 4 and 5, resulting in increased ROS production, degradation of cAMP and cGMP, and vasoconstriction [79,80]. Moreover, diabetic Ecs have impaired responses to EDHF [81].

Other dangerous changes may be the extreme alpha-adrenergic coronary vasoconstriction described in metabolic syndrome and the activation of the renin–angiotensin–aldosterone system increasing angiotensin II-mediated coronary vasoconstriction, as observed in hypertensive patients [82,83].

### 2.4. Role of Mitochondrial Dysfunction in Diabetic CMD

Mitochondria constitute only 10% of the volume of Ecs, where primarily the anaerobic metabolism supplies ATP under physiologic conditions. Nevertheless, mitochondrial dysfunction represents a pivotal defect in the development of CMD in diabetes due to hyperglycemia-induced alterations of both the metabolism and the dynamics of these organelles in the endothelium [84,85].

Under hyperglycemic stress, EC mitochondria reduce the oxidation of glucose and promote that of fatty acids, resulting in the lowering of the ATP/ADP ratio and oxygen consumption rate and damage or uncoupling of the mitochondrial oxidative phosphorylation system, which stimulates ROS production [32]. Supraphysiologic ROS levels activate the polyADP-ribose polymerase pathway, which inhibits, through ribosylation, the glycer-aldehyde-3-phosphate dehydrogenase, causing the buildup of glycolytic intermediates and their shunting into the above-described alternative pathways, which accelerates oxidative stress in a detrimental vicious circle [32,86]. Electron microscopy of the coronary microvascular Ecs of patients scheduled for coronary artery bypass grafting revealed large vacuoles and swollen mitochondria in diabetic subjects with poor glycemic control [87]. Animal experiments demonstrated disruption of endothelial mitochondrial dynamics by hyperglycemia [88]. In particular, diabetic EC manifests an imbalance between mitochondrial fission, a process responsible for the elimination of damaged and dysfunctional organelles, and mitochondrial fusion, which sets up a dynamic network in order to respond to metabolic changes, further contributing to ROS production and endothelial dysfunction [84]. Hyperglycemia represses the protective mechanism of mitophagy through altered homeostasis of defective mitochondrial fragments and release of apoptogens to initiate caspase-dependent apoptosis in Ecs. In addition, defective mitochondrial biogenesis may impair the replacement of damaged mitochondrial populations in diabetes [32].

### 2.5. Oxidative Stress and Inflammation in Diabetic CMD

The mechanisms leading to CMD are complex and not entirely clear. However, oxidative stress caused by ROS overproduction in both the cytoplasm and mitochondria, together with inflammatory response, are considered to be key pathogenic processes exacerbating one another in the development of CMD [89]. As a confirmation, in a series of experiments on diabetic or prediabetic rodents, upregulation of SOD and phosphorylated eNOS, inhibition of NOX and mitocondrium respiratory chain, administration of an O^−2^ scavenger or of TNF-α and IL-6 neutralizing antibodies, were all associated with the improvement of endothelial dysfunction [64,90,91,92].

Under normal conditions, low-level generation of ROS has an important physiologic role in maintaining healthy endothelium-dependent vasomotor function. For instance, hydrogen peroxide released by coronary endothelial cells in response to shear stress and to contracting myocardium in the setting of an increased myocardial oxygen demand, was shown to induce a NO-independent arteriolar vasodilation via both endothelium-dependent and -independent pathways [93]. Endogenous ROS may play a protective role in coronary endothelial homeostasis by inducing AMP-activated protein kinase (AMPK)-mediated activation of eNOS, NO synthesis, and endothelium-dependent vasodilation [94]. Similarly, an increase of NOX expression may improve NO synthesis and endothelium-dependent coronary vasodilation [95]. Otherwise, the excess ROS generation in disease conditions is associated with endothelial dysfunction and vascular remodeling.

In the vasculature, the major sources of ROS are NOX, xanthine oxidase (XO), eNOS uncoupling, and the mitochondrial electron transport chain, with O^−2^ as the predominant effector of oxidative stress [96]. Among these, NOX and mitochondria are two mutually related players, as the activation of NOX triggers the phosphorylation of the pro-apoptotic protein p66Shc and its translocation within the mitochondria, where it further enhances ROS generation. In turn, p66Shc activation stimulates the activity of NOX, thus generating a vicious cycle of ROS augmentation [97,98,99].

Apart from enhanced production, accumulation of ROS may also depend upon the impairment of scavenging pathways due to reduced expression of antioxidant enzymes, such as SOD, catalase, and dehydrogenases [25].

On the other hand, several findings indicate that inflammation may disrupt endothelial function by increasing oxidative stress. C-reactive protein, a biomarker of inflammation and CV disease, has been shown to inhibit NO-mediated coronary arteriolar dilation through the production of O^−2^ from NOX via p38 kinase activation, and endothelium-dependent prostacyclin-mediated dilation, by inhibiting prostacyclin synthase [100,101].

T cells and macrophages, as producers of the pro-inflammatory cytokines TNF-α and interleukins (ILs), are central factors in endothelial dysfunction and vascular remodeling [25]. TNF-α may deplete NO bioavailability through two major mechanisms: the reduction of eNOS mRNA stability and expression observed in HUVECs, and the reduction of NO through its ability to enhance ROS generation [102]. This latter occurs potentially either via an immediate activation of NOX, or via impairment of the mitochondrial respiratory chain [103]. In porcine coronary arterioles, TNF-α inhibited endothelium-dependent NO-mediated dilation by ceramide-induced activation of JNK and subsequent production of O^−2^ via XO [104,105]. Along with TNF-α, IL-6 contributes to inflammatory injury of the coronary endothelium in diabetes [106].

Specularly, activation of the AGE/RAGE and ROS pathways, angiotensin II, and ET-1 in diabetes and in hypertension culminates in high NF-κB levels. These factors contribute to microvasculopathy progression by increasing the expression of pro-inflammatory cytokines and of intercellular adhesion molecule-1 (ICAM-1) and vascular cell adhesion molecule 1 (VCAM-1), two factors strongly associated with the phenotype switching and proliferation of VSMCs and with vascular remodeling [25,107].

### 2.6. Remodeling of Coronaric Microvessel and Myocardium in Diabetes

In the myocardium, as in other tissues, the impairment of microvascular function evolves at later stages of diabetic disease into structural change, in order to restore normal vascular wall tension [108].

The anatomical abnormalities of CMD are extensive and are mainly represented by thickening of the capillary basement membrane and of the arteriole wall, resulting in luminal narrowing, perivascular fibrosis with focal constriction, and capillary rarefaction. All these factors increase coronary microvascular resistance, reduce flow reserve, and may produce regional ischemia, even in the absence of epicardial stenosis [109,110].

Interestingly, in myocardial tissue specimens collected from patients with end-stage HF, with or without diabetes at the time of transplantation, capillary rarefaction and pericyte loss were observed only in diabetic explants. Similar alterations were detected in the hearts of diabetic pigs aged 5 months [111].

The same pathogenic mechanisms are in play for both functional and morphological alterations of CMD, these being the persistence of NO deficit, ROS excess, and inflammation, responsible for the alterations to the cellular and non-cellular constituents of the microvasculature wall. In addition to preservation of vascular tone homeostasis, NO is an important regulator of vascular remodeling through direct anti-fibrotic effects, resulting from the inhibition of VSMC proliferation and extracellular matrix protein expression [112]. Instead, NO reduction impairs cGMP and transforming growth factor-β (TGF-β) functions and favors the conversion of ECs into mesenchymal cells that can give rise to fibroblasts [113,114,115]. An excess of ROS induces microvascular remodeling through mediation of growth factors, such as platelet-derived growth factor (PDGF) and TGF-β [116,117]. Impaired autophagy and enhanced apoptosis in CMECs may contribute to the capillary rarefaction observed in CMD [118]. Angiotensin II is an important regulator of vascular remodeling via VSMC hypertrophy and hyperplasia, and the AT1 receptor-mediated activation of VSMCs through pro-inflammatory signaling may participate in the remodeling observed in T2DM [119,120].

Based on Poiseuille’s Law, small changes in blood vessel diameter may have important effects on flow, and these structural modifications impair coronary blood flow and coronary flow reserve, suggesting that microvascular remodeling accounts for at least some of the deleterious ischemic events in T2DM and metabolic syndrome.

In mouse and swine models of T2DM or metabolic syndrome, coronary resistance microvessels undergo inward hypertrophy, leading to a decrease in lumen diameter and an increase in wall thickness and the wall-to-lumen ratio [121,122]. Alternatively, coronary microvasculature from mice with streptozotocin (STZ)-induced T1DM did not exhibit any structural alterations [123]. This difference depends upon the distinct pathophysiology of T2DM, which is preceded by a pre-diabetic phase characterized by early metabolic imbalance, increased insulin resistance, and a slight increase in fasting glucose levels that already initiates CMD. In contrast, T1DM-associated CMD occurs a while after the onset of diabetes and is strongly correlated with disease duration [124].

### 2.7. Other Mechanisms of CMD: Hemodynamic Forces, Epigenetics and microRNAs

The endothelial layer of the coronary microvasculature is directly exposed to hemodynamic forces such as arterial pressure, which is frequently elevated in diabetes, and shear stress, both of which may induce vasomotor changes and remodeling [125]. High blood pressure elicits constriction of isolated small coronary arteries and arterioles in rats, which may protect the distal arterioles and capillaries and prevent oedema from high hydraulic/filtration pressure [126,127]. On the other hand, this vasoconstriction increases the blood flow velocity and shear stress on the endothelium of upstream large vessels and at the bifurcation level [128].

In hyperglycemia and insulin resistance, epigenetic disruption may affect microvascular endothelial function [89]. One commonly studied epigenetic change is associated with the p66Shc gene activity involved in increased ROS production. In the setting of intensive glycemic control as well as in the vascular hyperglycemic memory, this phenomenon is responsible for the persistence of apoptosis and reduced NO bioavailability [129,130,131]. In addition to the modifications to chromatin, several non-coding RNAs (miRNAs) are implicated in microvascular dysfunction through the targeting of relevant pathways involved in endothelial cell and VSMC damage in diabetes [132]. A direct link between hyperglycemia and upregulation of miR-92a, associated with inflammatory phenotype and impaired angiogenesis, has been demonstrated in cardiac microvascular ECs from human T2DM patients and in non-diabetic ECs cultured with higher glucose levels [133]. A very recent study on mouse cardiac microvascular ECs identified a novel molecular mechanism associated with the upregulation of miR-27a-3p. This mechanism was responsible for the development of hyperglycemia-induced metabolic memory and persisted even after the culture medium had been switched back to the normal glucose level for a long time [134].

## 3. Therapeutic Management of CMD in Diabetes

Mounting evidence confirms the key role of CMD in a group of CV pathologies that frequently affect diabetic patients, such as ischemic heart disease.

In the setting of significant epicardial artery stenosis, the additional resistance from coexisting CMD further limits maximal coronary blood flow [135]. Notably, CMD, with or without vasospastic angina, is one of the major endotypes of myocardial ischemia without obstructive CAD, which, when present, results in a significantly worsened prognosis with a higher risk of major adverse CV events at 5 years [136]. In a recent retrospective cross-sectional study involving an unselected population of T2DM patients experiencing chest pain and non-obstructive CAD, 72.1% of the 129 studied diabetic patients had an endothelial-dependent or -independent CMD, which was associated with a linear increase of HbA1c amongst women but not men [137].

The failure of effective microcirculatory perfusion after successful epicardial revascularization (“no-reflow” phenomenon), represents a poor prognostic factor associated with adverse ventricular remodeling, HF, and mortality [138,139,140,141]. No-reflow appears to be significantly correlated with admission glucose levels and higher in-hospital peak glycemia, and a recent study reported a positive association between the phenomenon and diabetes duration and higher preoperative blood glucose level [142,143,144]. The phenotype of HFpEF typically affects people suffering from diabetes and obesity, two morbidities sharing low-grade systemic inflammation that may trigger dysfunction and remodeling of both the microvasculature and myocardium and which likely represent the core defect in the intricate and not-yet fully understood pathophysiology of this type of HF [145,146,147]. Tromp et al. found microvascular disease to be more common in diabetic persons with HFpEF than those with HFrEF, suggesting that HFpEF could be a possible clinical manifestation of CMD in diabetes [148].

These observations highlight the crucial role of CMD in CV prognosis in diabetes. Nevertheless, effective strategies to prevent the progression of this complication are lacking, and to date, no specific treatment has been validated by large-scale randomized clinical trials (RCTs).

### 3.1. Correction of CV Risk Factors

At the moment, the therapeutic management of patients with CMD is primarily aimed at aggressive correction of CV risk factors, fundamentally motivated by the great prevalence of coronary artery disease (CAD) in patients with CMD [149]. Among the typically taken lifestyle measures, weight control represents a first priority due to the strict association of diabetes with obesity. There is substantial recognition of the impact of obesity-associated low-grade systemic inflammation on heart microvascular function and of the beneficial effect of body weight reduction on endothelial function, which may manifest as early as 1 week after the initiation of dietary intervention [150,151]. A recent study confirmed these data, demonstrating that a short-term reduction of BMI from 31.8 to 27.5 in middle-aged obese subjects improved microvascular endothelial function [152]. The benefits to coronary flow reserve and peripheral vascular function attained with aerobic interval training was of comparable substance to those of weight loss in revascularized obese CAD patients [153]. In an interesting study involving 827 patients with normal perfusion at evaluation for CAD with PET cardiac stress who were followed for a median of 5.6 years for CV events, CMD was independently associated with elevated BMI and adverse outcomes, resulting as a better discriminator of risk than BMI and other traditional risk factors [154]. There is documentation of improved CMD after bariatric surgery [155,156].

### 3.2. Pharmacological Options

The benefits obtained through pharmacological therapy in CMD are poorly documented, and at any rate, generally not specifically evaluated in diabetic people.

There is some evidence of late improvement of CMD by fluvastatin that occurs after six months of therapy [157]. Instead, in a recent RCT with rosuvastatin, a treatment of equal duration did not improve microvascular function in women with chest pain and no obstructive CAD [158]. Third-generation beta-blockers and dihydropyridine-type calcium channel blockers were able to ameliorate endothelial function in addition to reducing myocardial oxygen demand and increasing diastolic perfusion time [135]. Inhibitors of the renin–angiotensin system may improve coronary microvascular function by blocking the powerful vasoconstrictor effects of angiotensin II, but they failed to lower overall morbidity or mortality in patients with HFpEF [159,160]. Likewise, sacubitril–valsartan did not significantly improve the rate of total HF hospitalizations and CV death in HFpEF patients with respect to valsartan alone [161]. Two RCTs of ranolazine in patients with CMD showed no significant benefit to symptoms or coronary microvascular function [162,163]. On the contrary, in a recent metanalysis of RCTs evaluating the effect of antianginal drugs (ranolazine, nicorandil, and ivabradine) on CMD in patients with non-obstructive CAD, only ranolazine improved the myocardial perfusion reserve index, whereas both ranolazine and ivabradine reduced angina [164]. Antithrombotic therapy deserves strong consideration due to the prothrombotic state and the formation of hyperreactive platelets characterizing diabetes and their role in the multifactorial pathogenesis of CMD, in addition to the strong association between atherosclerosis and CMD [165]. Aspirin may be prescribed for the primary prevention of CVD in patients with diabetes at a high risk of CV events but is not in those at moderate or low risk due to an increased risk of major bleeding which largely offsets the CV benefits [166,167]. Instead, antiplatelet drugs provide a fundamental pharmacological strategy in secondary CV prevention in diabetes by utilizing aspirin or a P2Y_12_ inhibitor or a combination of two drugs, depending on the clinical context, taking into account that the superior efficacy of more potent antithrombotic approaches most often occurs at the expense of increased bleeding [168].

Notably, diabetic patients have poorer outcomes than their non-diabetic counterparts after percutaneous coronary intervention [169,170]. Among the associated causes is the higher prevalence of periprocedural myocardial infarction which involves a series of factors among which is chronic microvascular dysfunction. New antiplatelet agents might play a protective role in this setting, particularly ticagrelor, which reduces the physiological clearance of adenosine. The increased concentration of this nucleoside may protect the myocardium from both ischemic and reperfusion injuries via its potent vasodilator effect, in addition to its anti-inflammatory and antiplatelet properties [171]. Interestingly, a more pronounced effect of adenosine on microcirculatory resistance seems to characterize obesity and diabetes [172]. In a study on patients with ST-elevated acute coronary syndrome, the myocardial microcirculation perfusion level was significantly higher in participants affected by diabetes treated with ticagrelor than those treated with clopidogrel [172,173].

Overall, the potential as an effective drug treatment for CMD is rather limited. Interestingly, the older antihyperglycemic drug metformin has shown that it could be a valuable therapeutic tool for endothelial protection in diabetic patients [174,175]. One positive turn for diabetic patients is that recent advances in glucose-lowering medications have provided a successful strategy for targeting CVD, obesity, and HF, even presenting a potential tool to correct CMD. Hereafter, we describe the available data in the literature related to this topic.

## 4. Glucagon like Peptide-1 (GLP-1) and GLP-1 Receptor Agonists (GLP-1 RAs)

GLP-1, a major incretin synthesized and secreted by intestinal L cells in response to nutrient ingestion, exerts glucoregulatory action via stimulation of glucose-dependent insulin secretion from pancreatic β-cells, inhibition of glucagon secretion from pancreatic α-cells, and deceleration of gastric emptying, attenuating postprandial hyperglycemia [176]. In patients with T2DM, the secretion of GLP-1 appears to be diminished, but its biological actions are largely preserved [159]. Therefore, GLP-1-based therapies including structural analogues of human endogenous GLP-1 (albiglutide, dulaglutide, liraglutide, and semaglutide) and exendin-based agents (efpeglenatide, exenatide, and lixisenatide), have emerged as major anti-hyperglycemic therapeutic options [177,178]. In a metanalysis of 14 clinical trials, Huthmacher et al. calculated that the reduction in HbA1c by GLP-1 RA was 0.5% with short-acting agents and 1.0% with long-acting agents, allowing more patients to achieve glycemic targets [179]. This potent glucose-lowering effect is achieved without increasing the risk of hypoglycemia.

### 4.1. CV Benefits by GLP-1 RAs

In addition to its glucose-lowering effects, large prospective outcome trials with GLP-1 RAs showed significant reduction of the risk of CV events and mortality in T2DM patients, particularly for liraglutide, semaglutide, albiglutide, and dulaglutide [180,181,182,183]. A recent meta-analysis including seven large-scale trials (ELIXA, LEADER, SUSTAIN-6, EXSCEL, Harmony outcomes, REWIND, and PIONEER-6) with a combined total of 56,004 participants, confirmed this benefit, showing a significant reduction of the composite endpoints of CV death, non-fatal stroke, and non-fatal MI by 12%, and of hospital admission for HF by 9% [184]. These results cannot be explained solely by lowered glucose levels and are independent of metformin use, as suggested by subgroup analyses [185,186]. Notably, drugs with greater half-lives/duration of action, such as liraglutide (11–15 h) and semaglutide (7 days), demonstrated superiority in CVD outcomes trials [187].

Based on the impressive results obtained from CV trials using these drugs, guidelines from the American Diabetes Association have undergone an epochal turning, because, without taking into account the level or individualized target of HbA1c, they recommend GLP-1 RAs for T2DM patients with established atherosclerotic CVD or at high risk thereof [188].

#### 4.1.1. Correction of Traditional Risk Factors for CVD

The mechanisms behind the potent CV protection provided by GLP1-RAs are still not completely clear. Apart from HbA1c, these drugs have been shown to modestly reduce systolic blood pressure, decrease small dense LDL particles, and to correct relevant CV risk factors associated with T2DM such as obesity [189,190]. A number of observational and interventional studies on glycemic reduction by GLP-1 RA in T2DM patients reported weight loss, and several studies confirmed this effect in obese subjects without diabetes. A greater impact on body weight is associated with the use of higher doses of semaglutide, which attain a significant weight reduction of 14.9% from baseline, compared with 2.4% with placebo, without the weight-reduction plateau noted with other antiobesity medications between 30 and 40 weeks [191,192]. Being expected to have a critical role in the management of obesity, GLP-1RAs have been approved by the FDA for chronic obesity management in non-diabetic people [193].

The weight loss induced by semaglutide does not result from an increase in energy expenditure, but rather from a reduction in energy intake determined by less appetite and food cravings, better control of eating, and lower relative preference for fatty, energy-dense foods [194]. These data are consistent with clinical studies using other GLP-1 RAs [195,196].

Previous human studies showed that by accessing specific areas of the brain relevant for appetite regulation, exenatide decreased food-related brain responses in T2DM patients and obese subjects, and similar findings were observed in mouse models of obesity treated with liraglutide [197,198,199]. In other animal investigations, the binding of GLP-1RAs in various hypothalamic sites induced a reduction of body weight by both regulating food intake and increasing energy expenditure through stimulation of the thermogenesis of brown adipose tissue and the browning of white adipose tissue [200].

#### 4.1.2. Direct Anti-Atherosclerotic Effects

The mechanisms underlying the cardioprotective effects of GLP-1 RAs are not explained by the mere modification of traditional risk factors.

In accordance with the wide expression of GLP-1R in tissues other than those in the gastrointestinal tract, including vascular endothelium and smooth muscle cells, a direct anti-atherosclerotic action has been described for these drugs [176,201]. Experimental studies have suggested a series of underlying mechanisms, such as modulation of metalloproteinases through the inhibition of AKT-Thr308 phosphorylation observed in coronary artery smooth muscle cells, prevention of Ang II-induced vascular smooth muscle cells’ proliferation and migration, suppression of macrophage foam cell formation, anti-inflammatory properties in ECs caused by downregulating the activation of NF-κB and the adhesion molecules ICAM and VCAM, and beneficial effects on endothelial function through nitric oxide-induced vasodilation and reduced oxidative stress [202,203,204,205,206,207]. In vitro studies on human ECs reported that GLP-1 attenuated reactive oxygen species-induced senescence in a receptor-dependent manner, and that liraglutide reduced the HG-induced oxidative stress and TNF-α-induced inflammation [208,209,210,211]. A recent study described multiple facets of the endothelial protection provided by liraglutide against the damage induced by oxidized LDL. In addition to mitigating the reduction of eNOS expression and NO release, the drug caused an amelioration of increased permeability, inhibition of vascular adhesion molecules’ expression, and prevention of monocytes’ adhesion to cultured ECs [212]. In experiments in ApoE-/-mouse models, liraglutide increased eNOS with significant improvement in endothelial function, and exendin-4 reversed the high-cholesterol diet-induced endothelial dysfunction through a GTP cyclohydrolase-1/tetrahydrobiopterin pathway [213,214]. In ex vivo studies on diabetic patients, exenatide increased eNOS activation and NO production in ECs and reduced HG- or lipid-induced endothelial dysfunction in arterioles, both through GLP-1R and AMPK activation [215]. Liraglutide ameliorated the stress to the endoplasmic reticulum and restored insulin-mediated e-NOS activation in ECs from T2DM patients [216]. Nyström et al. first showed that GLP-1 ameliorated endothelial dysfunction in T2DM patients with established CAD [217]. In individuals with T2DM diabetes, intravenous exenatide increased fasting endothelial function, and subcutaneous exenatide increased postprandial endothelial function independently of reductions in plasma glucose and triglycerides [215]. Other human studies in T2DM patients described a reduction of oxidative stress by using liraglutide [218,219].

### 4.2. Effects of GLP-1 RA on Coronary Microvasculature

Vascular endothelium expresses GLP-1 R that, likewise to insulin receptor, induce a vasodilatation of conduit and resistance arteries and of microvasculature. Unlike insulin that acts trough the PI3-kinase pathway, the activation of GLP-1 R elicits endothelium mediated NO production through the cAMP-PKA signaling [220,221].Some studies report expression of GLP-1Rs in VSMCs but not in coronary ECs [222,223]. Other investigators indicate beneficial effects of GLP-1 on myocardial and endothelial cells mediated via GLP-1R-independent pathways [222,223,224]. Nevertheless, GLP-1-based therapy may be considered a potential regimen for the correction of cardiac microvascular injury in diabetes, as suggested by a series of preclinical and clinical findings (Table 1).

#### 4.2.1. Preclinical Studies

GLP-1 treatment could protect ECs against oxidative stress-induced autophagy and inflammation, and in a very recent study on HUVEC, liraglutide improved EC function through suppression of the PINK1/Parkin-mediated mitophagy [210,212,225].

Some experiments have been designed to specifically determine the effects of GLP-1 on cardiac microvessels in diabetes. STZ-induced diabetic rats received a 12-week treatment with vildagliptin (an inhibitor of dipeptidyl peptidase-4 inhibitor, the enzyme that rapidly degrades the native GLP-1) or exenatide (a GLP-1 analog that shares many biological functions with GLP-1). Both treatments significantly preserved cardiac micro-vascular integrity and attenuated the diffusion of lanthanum nitrate across ECs, indicating protective properties towards coronary microvessel barrier function with improved cardiac glucose metabolism and diastolic function [226]. To characterize the underlying molecular mechanisms, in in vitro experiments on CMECs cultured under HG conditions, showed that GLP-1 decreased the HG–induced ROS production and the apoptotic index through the inhibition of activation of the Rho/ROCK pathway, a key mediator for oxidative stress-induced cell injury [226].

H/R injury is an event which occurs primarily due to the oxidative stress of CMECs [227]. Zhang et al. induced H/R injury in CMECs to explore the mechanisms involved and to identify, among the various sources of ROS, the role of XO, an enzyme primarily located on the luminal surface of the microvascular endothelium of many organs, the heart included [228]. They demonstrated that the H/R-induced oxidative damage in CMECs was caused by Ca^2+^ overload that increased XO-mediated ROS release, and that liraglutide pre-treatment could suppress such injury by stimulating the PI3K/Akt/surviving pathways [229].

A more recent study investigated whether treatment with liraglutide improved endothelial vasodilator function across the coronary microcirculations and prevented cardiac remodeling in obese Zucker rats [230]. To exacerbate their metabolic syndrome, the animals were chronically exposed to a high Na^+^ diet that previous studies associated with cardiomyopathy and vascular injury [231]. After an 8-week period, liraglutide treatment downregulated NOX1 mRNA and reduced ET-1 protein expression, thus restoring the balance of tonic ET-1/NO production and improving myocardial perfusion in the small arteries and arterioles. In addition, liraglutide significantly attenuated the expression of proinflammatory and profibrotic biomarkers (NF-κB, CD68, IL-1β, TGF-β1, and osteopontin) and of nitrotyrosine in comparison to untreated rats. Lastly, liraglutide normalized the cardiomyocyte cross-sectional area and partially reduced the development of perivascular fibrosis in association with reduced expression of protein markers of remodeling, including osteopontin and TGF-β1 [230].

Taken together, these results suggested that liraglutide treatment restored the endothelial function of coronary arterioles by increasing NO bioavailability as well as reducing the induction of inflammation.

A histochemical study demonstrated that the classic morphological findings of diabetic cardiomyopathy observed in adult male albino rats with STZ-induced diabetes were markedly improved by liraglutide treatment. The treatment nearly preserved normal myocardiac structure and significantly protected against myocardiac inflammation and fibrosis. In addition, liraglutide decreased TNFα expression and increased VEGF protein and the density of coronary arteriolar vasculature, as indicated by a significant increase in α-smooth muscle actin [232].

Some authors have evaluated the effects of GLP-1 in post-resuscitation syndrome, a very deadly condition whose major component is myocardial microcirculatory dysfunction [233]. The findings, obtained in a swine model of prolonged ventricular fibrillation, suggested that post-resuscitation treatment with GLP-1 exerts positive effects on coronary microcirculation elevated by adenosine-stimulated CFR, although without subsequent improvements to LV function [234]. Since adenosine may act directly on vascular smooth muscle, these experiments did not determine the effects of GLP-1 on the coronary microvascular endothelium, the tissue most injured during reperfusion [235]. In a subsequent investigation in the same animal model, the authors found that the continuous intravenous infusion of GLP-1 for 4 h after cardiac arrest and resuscitation preserved coronary microvascular endothelial function, likely through an antioxidant effect as indicated by the decreased production of 8-iso-PGF2α in the heart [236].

#### 4.2.2. Human Studies

A study conducted over ten years ago hypothesized that GLP-1cardioprotection could depend upon increased myocardial glucose availability and utilization. Exenatide, a synthetic GLP-1 receptor agonist, was acutely administered to eight male subjects with suboptimally controlled non-insulin-treated T2DM and without CAD in order to evaluate myocardial glucose uptake and MBF using PET during a pituitary-pancreatic hyperglycemic clamp with 18F-fluorodeoxyglucose and 13N-ammonia as tracers. It was demonstrated that acute treatment with a GLP-1 analog did not alter myocardial glucose uptake but resulted in MBF improvement by 24% [237]. Since exenatide does not seem to have any acute hemodynamic effects on heart rate or blood pressure in humans and the rise in MBF was not accompanied by increases in catecholamines, direct action through a receptor in the myocardium was speculated [238].

This positive effect on MBF was replicated in 26 healthy young volunteers receiving infusions of GLP-1 to raise plasma concentrations to their postprandial levels. After 150 min, contrast-enhanced echocardiography with injection of octafluoropropane gas-filled lipid microbubbles to trace the cardiac microvasculature revealed substantial microvascular recruitment with enhanced cardiac perfusion as indicated by an increase in microvascular blood volume by 57% and in MBF by 47% with a concomitant decrease in microvascular flow velocity [239]. A small double-blind randomized cross-over study performed in 12 obese participants with normal glucose tolerance, found no effects of intact GLP-1 administered with a DPP-4 inhibitor on coronary microvascular function evaluated by Doppler CFVR or on peripheral endothelial function evaluated by flow mediated dilation [240]. In a similar study, fifteen obese adults received intravenous infusion of either saline or GLP-1 with or without a superimposed euglycemic insulin clamp. In addition to confirming a significant insulin resistance in the conduit artery and in the skeletal and cardiac muscle microvasculature associated with obesity, the study convincingly demonstrated that the vasodilatory action of GLP-1 was blunted in the conduit artery but preserved in the skeletal and cardiac muscle microvasculature, as evaluated by contrast-enhanced echocardiography [221]. The microvascular recruitment induced by GLP-1 was independent of insulin, as GLP-1 infusion only slightly raised plasma insulin concentrations, and insulin infusion alone failed to dilate skeletal and cardiac muscle microvessels, suggesting the presence of insulin resistance at this level. Notably, in this study a DPP-4 inhibitor was not associated with GLP-1 administration.

Other investigators evaluated the effects of GLP-1 in patients awaiting PCI for stable angina. Clarke et al. demonstrated a microcirculatory vasodilator effect after peripheral GLP-1 administration by measuring the post-PCI coronary blood flow by a pressure-flow wire in 21 patients, even if GLP-1R expression in coronary vascular endothelium or smooth muscle cells was not found with immunohistochemistry [241]. The authors documented a positive staining of GLP-1R on ventricular myocytes and a transmyocardial gradient of GLP-1 but not of its major metabolite indicating a myocardiocyte GLP-1 extraction. Based on these data, they suggested a direct action of GLP-1 on cardiomyocytes promoting enhanced contractility, in accord with the observed augmentation of LV function, and an indirect influence on coronary microvascular flow through ventricular–coronary interactions.

This finding supported a direct action of GLP-1 on cardiomyocytes promoting enhanced contractility, in accord with the observed augmentation of LV function, and with an indirect influence on coronary microvascular flow through ventricular–coronary interactions. In this regard, it is known that coronary perfusion and myocardial contractility are linked by several factors in order to match supply and demand, and that physical forces from increased inotropy and improved lusitropy open the adjacent microvessels [242]. In turn, increased microvascular volume can promote muscle contractility by opening stretch-activated calcium channels, resulting in increased intramyocardiocyte Ca^2+^ transient and Ca^2+^ sensitivity and higher muscle contractility (Gregg effect) [242]. A subsequent study performed by the same research group in 41 patients who had received a PCI for stable angina investigated whether the coronary microvascular vasodilatation induced by GLP-1 infusion was mediated by adenosine. Since the GLP-1-induced vasodilation was not abolished by theophylline, and GLP-1 did not increase adenosine levels, an adenosine-independent mechanism behind the GLP-1 coronary vasodilatation was suggested [243].

Some small trials have evaluated the effects of chronic GLP1-based therapy and they have obtained mostly negative results.

Chen et al. observed that a short 7-day course of liraglutide in STEMI patients treated with primary percutaneous coronary intervention (PCI) was associated with a tendency for a lower rate of no-reflow and a mild improvement in LVEF at 3 months [244].

A randomized, single-blind, crossover study with 1.2 mg liraglutide evaluated the changes in CFVR after 10 weeks of treatment versus no treatment in 20 T2DM patients with no history of CAD, myocardial ischemia, or HF [245]. Although liraglutide was associated with weight loss, lower systolic blood pressure, and improved HbA1c, only a borderline non-significant effect on coronary microvascular function was registered with Doppler-flow echocardiography during dipyridamole-induced stress. The authors concluded that further long-term studies, preferably in patients with more substantially affected microvascular function and using a higher dosage of GLP-1 analogues, were needed to confirm these findings.

In an open-labeled paralleled study, 31 newly diagnosed T2DM subjects were given to lifestyle intervention with or without exenatide. After a 12-wk treatment, CFVR assessed with TTDE was significantly improved by GLP-1 agonist, in connection with a remarkable decrease in the serum levels of soluble ICAM-1 and VCAM-1 [246]. In the same study, in vitro experiments in cultured primary HUVECs, exendin-4, a form of exenatide, induced a dose-dependent increase in NO production, eNOS phosphorylation, and the level of GTP cyclohydrolase 1, the rate-limiting enzyme in de novo biosynthesis of BH4, an essential cofactor for eNOS. These effects were abolished by the addition of the GLP-1R antagonist exendin, GLP-1R siRNA, adenylyl cyclase inhibitor SQ-22536, AMPK inhibitor compound C, and PI3K inhibitor LY-294002 to the culture. The authors concluded that exenatide significantly improved coronary endothelial function in patients with newly diagnosed T2DM, and that this effect was likely mediated primarily through activation of AMPK and the PI3K/Akt pathway in a GLP-1R/cAMP-dependent manner [246].

In a double-blind trial, 36 non-diabetic patients suffering from clinically stable HFrEF were randomized to the liraglutide or placebo groups. After 24 weeks, no improvements in myocardial glucose uptake, MBF, or MFR were registered [247].

A limitation of these human studies is the small sample size, which could justify the discrepancy in the results on its own. Other grounds may be the age and BMI of patients and their degree of baseline microvascular dysfunction. Importantly, during the infusion of intact GLP-1, a high concentration of GLP-1 metabolite will be present in the circulation due to rapid transformation by the ubiquitous enzyme DPP-4. The influence on vascular actions from metabolite changes caused by this enzyme must be taken into account [224].

**Table 1:** Effects of GLP-1 and GLP-1 RAs on the coronary microvascular compartment.

Preclinical Studies						
Experimental Model	Treatment	Duration of Treatment	Effects	Suggested Mediating Mechanisms		Ref.
STZ-induced diabetic rats	exenatide or vildagliptin	12 weeks	protection of endothelial barrier function by both drugs (at transmission electron microscopy)			[226]
HG-cultured CMECs	Incubation with GLP-1		↓ ROS production and apoptotic index	inhibition of Rho though a cAMP/PKA-mediated pathway		[226]
H/R injuried CMECs	liraglutide 12 h before the induction of hypoxia		suppression of XO-mediated ROS release	stimulation of PI3K/Akt/surviving pathways		[229]
Zucker obese rats at high Na^+^ diet	liraglutide	8 weeks	restoration of ET-1/NO balance (↓ nitrosative stress and proinflamm./profibrotic markers)			[230]
STZ-induced diabetic rats	liraglutide	6 weeks	↓ TNF-α ↑ VEGF and α-smooth muscle actin			[232]
swine model of ventricular fibrillation	GLP-1	4-h infusion 1 min after resuscitation	↑ adenosine-stimulated CFR (by intracoronary Doppler flow)			[234]
swine model of ventricular fibrillation	GLP-1	4-h infusion 1 min after resuscitation	preserved endothelial function ↓ of oxidative stress (↓ 8-iso-PGF_2α_)			[236]
**Clinical Studies**						
**Experimental model**	**Treatment**	**Duration of treatment**	**Effects**	**Suggested mediating mechanisms**	**Diagnostic modality**	**Ref.**
15 obese people	GLP-1 +/− euglyc. insulin clamp	150-min infusion ≥120 min	↑ MBF by ~40% Independently of insulin	preserved recruitment of coronary microvasculature	myocardial contrast echocardiography	[221]
8 non-insulin treated T2DM pts without CAD	exenatide	IV infusion	↑ MBF by 24% (no change of myocardial myocardial glucose uptake)	direct action on a myocardial receptor (?)	^13^N-ammonia PET with hyperinsulinemic clamp	[237]
26 healty young people	GLP-1	150-min infusion	↑ MBF by 47%	recruitment of coronary microvasculature	myocardial contrast echocardiography	[239]
12 normo-tolerant obese people	GLP-1 + sitagliptin	120-min infusion	no variation of CFVR	no direct effect of intact GLP-1 on coronary microvessels	TTDE	[240]
21 patients awaiting PCI for stable angina	GLP-1 (n.10) or saline (n. 11)		post-PCI vasodilation of coronary microvessels	direct actions on cardiomyocytes	pressure-flow wire	[241]
41 patients awaiting PCI for stable angina	GLP-1 (n. 10) saline (n. 11) GLP-1 + Theophylline (n. 10) Theophylline (n. 10)	acute infusion post successful PCI	post-PCI vasodilation of coronary microvessels	adenosine-independent mechanism	pressure-flow wire	[243]
STEMI patients treated with PCI	liraglutide	7 days (started 30 m’ before PCI)	tendency for a lower rate of no-reflow		TTDE	[244]
20 T2DM patients without heart disease	liraglutide	10 weeks	borderline improvement of CFVR		TTDE with dipyridamole stress	[245]
31 newly diagnosed T2DM subjects	exenatide	12 weeks	improved CFVR ↓ serum ICAM and VCAM	activation of AMPK/PI3K/Akt pathway in a GLP-1R/cAMP dependent manner (observed in exenatide-treated HUVEC)	TTDE	[246]
36 non-diabetic patients with stable HFrEF	liraglutide (n. 18) or placebo (n. 18)	24 weeks	no variation in MBF or MBF reserve		^15^O-H_2_O PET	[247]

## 5. SGLT2 Inhibitors (SGLT2-Is)

SGLT2 is a carrier mainly expressed in the kidneys which is responsible for about 90% of the renal glucose uptake. It translocates glucose and Na^+^ across the apical membrane of tubular cells utilizing the downhill Na^+^ gradient provided by the activity of the Na^+^/K^+^ -ATPase. In contrast, SGLT1 only makes up around 10% of renal glucose uptake and is predominantly expressed in various tissues, such as those from the small intestine and brain, in addition to kidney [248].

By selectively inhibiting renal glucose reabsorption, SGLT2-Is (also called gliflozins) have emerged in the last decade as glucose-lowering drugs, producing appreciable improvements to glycemic control through a mechanism of action totally independent from insulin and related to the glucose amount that is filtered daily by the glomerulus, reaching the proximal tubule [249]. As largely demonstrated, SGLT2-Is produce a reduction in HbA1c of 7–10 mmol/mol (0.6–0.9%) in diabetic subjects without determining hypoglycemia. [250].

### 5.1. CV Benefits by SGLT2-Is

The inhibitors of the SGLT2 carrier possess unambiguous CV pleiotropic benefits that go beyond solely anti-hyperglycemic properties, and this is supported by large RCTs involving different gliflozins in both diabetes mellitus and HF, independently of each other disease [251,252,253,254,255,256]. Beyond the noticeable CV-beneficial effects of SGLT2-Is, reduced progression of chronic kidney disease was reported in T2DM patients along with an improvement of HF patients’ outcomes, regardless of diabetes, thus making it dutiful to include the use of gliflozins for these subsets of patients in the most recent guidelines [188,257].

Apart from hyperglycemia, gliflozins have been reported to ameliorate other modifiable CV risk factors through mechanisms that are not yet fully understood. Alone or in combination with other antihypertensive drugs, they demonstrated a modest reduction of both systolic and diastolic blood pressure [258]. The effects on the lipid profile of patients treated with gliflozins are not clear. In the main trials, it seems that this class of drugs causes a modest increase in both LDL and HDL cholesterol (about 4 mg/dL compared to placebo). However, in some recent experiments, these results have not been confirmed, and a slight reduction in both triglycerides and LDL cholesterol has been highlighted [259].

More interestingly, from the first week of treatment, SGLT2-Is can already lead to a reduction in body weight of about 1% to 3% from baseline. The effect is only partly attributable to the loss of glucose through the urine (300 kcal/day) which, besides, seems to be attenuated by reflex compensatory hyperphagia. Indeed, it appears likely that it is associated with early and rapid body water and fat loss up to around 2 months, followed by a slower rate of sustained decrease in both visceral and subcutaneous fat, ultimately reaching a plateau after 6 months and remaining stable over time. The fat loss seems to be generated by SGLT2-I modulation of fatty acids metabolism and stimulation of lipolysis with associated ketogenesis and fatty acid oxidation [260]. On the other hand, the decrease in extracellular fluid and plasma volume by gliflozins induces a reduction of afterload and preload, thus attenuating HF congestion [261]. Several preclinical studies have reported that gliflozins may act on systemic inflammation and oxidative stress, two factors notoriously associated with endothelial degeneration and atherogenetic processes [262]. The pathophysiological mechanisms are still debated and include multiple favorable actions on the vasculature, such as direct endothelium-independent vasorelaxation, reduction of vasoconstrictive substances, improvement of endothelial dysfunction, amelioration of atherosclerotic lesions, and lowered arterial stiffness. All these mechanisms were extensively studied in a recent review paper of our group [263].

### 5.2. Effects of SGLT2-Is on Coronary Microvasculature

SGLT2 has not been discovered in mammalian hearts or CMECs, and only some studies have reported the presence of small amounts of SGLT2 protein in ECs [264,265,266,267]. Nevertheless, based on literature data, a way in which SGLT2-Is may provide heart benefits could be the improvement of coronary microvascular function (Table 2).

#### 5.2.1. Preclinical Studies

Adingupu et al. tested the hypothesis that SGLT2-I empagliflozin could protect coronary microcirculation in a mouse model of metabolic syndrome and pre-/early diabetes with the peculiarity of developing coronary microvascular dysfunction but not atherosclerosis [268]. The advantage of this model of leptin-deficient obese mouse is that the CFVR assessed by TTDE may be considered a measure of microvascular function [269]. Ten weeks of empagliflozin treatment resulted in a significant improvement of CFVR and of fractional area change, an LF systolic index of contractility [270]. Based on the knowledge from humans that CFVR and cardiac systolic function are powerful predictors of cardiovascular outcome, the authors underlined the clinical relevance of their findings [271,272]. Regarding the involved pathophysiologic mechanisms, the lack of a detectable difference in CD31 staining did not suggest a recovery of structural capillary rarefaction. Instead, L-arginine and the L-arginine/ADMA ratio were higher in empagliflozin-treated mice compared to untreated, a result pointing to NO-dependent improvement of endothelial function as the relevant pathway for the amelioration of microvascular function [265]. This speculation was supported by a subsequent study providing the first evidence that the improvement of cardiac remodeling by empagliflozin observed in rats with and without diabetes subjected to ligation of the left anterior descending coronary artery was linked with the upregulation of cGCH1 and then with the activation of eNOS. The result was confirmed in a cardiomyocyte biomechanical stretching diabetic model, wherein the silencing cGCH1 blocked the preventive effect of empagliflozin on oxidative stress development [273].

The mediating role of NO in the amelioration of coronary microcirculation by SGLT2 inhibition was reinforced by experimentations conducted in vitro by Juni et al. [274]. The study aimed to establish an eventual direct causal effect of cardiac microvascular endothelial dysfunction on the alteration of myocardial contraction and relaxation, and to evaluate the impact of empagliflozin on the crosstalk between CMECs and cardiomyocytes in the pathogenesis of HFpEF. In a co-culture model that combined human CMECs and adult rat cardiomyocytes, it was shown that CMECs exerted a direct positive effect on cardiomyocyte function. This action was mediated by endothelial-derived NO and lost after preexposure of CMECs to TNF-α or IL-1b, therefore likely linked to the correction of NO scavenging by cytoplasmic and mitochondrial ROS. This result corresponded to an in vitro investigation on human amniotic epithelial cells (HAECs) and HUVECs, reporting that TNFα significantly elevated ROS levels and that treatment with empagliflozin and dapagliflozin restored NO bioavailability via the complete inhibition of the TNFα-induced upregulation of intracellular ROS levels, rather than via eNOS phosphorylation/expression [275]. In the study by Juni et al., empagliflozin counteracted the TNF-α-mediated impairment of CMEC–cardiomyocyte interaction through reduction of mitochondrial ROS levels, prevention of cytoplasmic ROS accumulation, and the enhancement of NO bioavailability within the CMECs and in conditioned medium, which resulted in the preservation of both the contraction and relaxation of cardiomyocytes [274]. In this connection, NO was shown to induce the soluble guanylate cyclase activity in cardiomyocytes and to increase cGMP levels leading to the activation of protein kinase G. This last, once activated, mediates the phosphorylation of several proteins, including troponin I, phospholamban, and titin, leading to enhanced reuptake of calcium into the sarcoplasmic reticulum and reduced cardiomyocyte stiffness [276]. These results are of interest for the pathogenesis of HFpEF where the inflammatory activation of CMECs might be a pivotal initial mechanism in the disruption of cardiac mechanical properties [277]. They also offer a pathophysiologic interpretation of the results of the EMPEROR-Preserved trial, whereby empagliflozin was reduced by ~21% the HF hospitalization and CV death in HFpEF patients with and without diabetes [278].

In another study, Juni et al. investigated the effects of uremic serum from patients with chronic kidney disease (CKD) on the CMEC–cardiomyocyte axis and endothelial control of cardiomyocyte function, by using, once again, the already developed co-culture system [279]. The study rationale was the significance of independent risk factors for CV complications of CKD, a condition characterized by plasma accumulation of uremic solutes and pro-inflammatory mediators promoting oxidative damage and the impairment of endothelial function [280,281]. The findings indicated that pre-exposure of CMECs to uremic serum impaired the endothelium-mediated enhancement of cardiomyocyte relaxation and contraction. The suggested pathophysiologic sequence was the induction of mitochondrial network fragmentation, namely mitochondrial fission, responsible for increased ROS production and intracellular accumulation, reduced endothelial NO bioavailability, and consequent impaired endothelium-mediated enhancement of CM relaxation and contraction. Empagliflozin reversed these detrimental events triggered by uremic serum on endothelium-to-cardiomyocyte crosstalk, primarily through the restoration of NO levels in ECs and cardiomyocytes [279]. The investigators confirmed this deleterious impact of whole uremic serum using indoxyl sulfate, an indolic uremic toxin originating from tryptophan metabolism with proven endothelial toxicity, notably via the induction of ROS and reduction of NO [282]. Moreover, they observed that the inhibition of mitochondrial oxidative radical production completely mimicked the beneficial influence of empagliflozin on endothelial-mediated cardiomyocyte function. Overall, these data provided a novel mechanism linking cardiac microvascular endothelial dysfunction to the pathogenesis of HF in CKD, and indicated, at least in vitro, that endothelial mitochondria were a major subcellular target of the observed benefits of empagliflozin.

The data produced by Juni et al. were in line with the suggestion made by other studies of the involvement of the mitochondria in the amelioration of coronary microvasculopathy associated with empagliflozin treatment [279]. Zhou et al. evaluated the effect of SGLT2 inhibition on the underlying mechanisms of diabetic cardiac microvascular injury with a focus on the mitochondria, using STZ-induced diabetic mice treated for 20 weeks with empagliflozin or with an inhibitor of mitochondrial fission. At the end of treatment, all mice were euthanized, and the hearts were collected for further experimentation [283]. The results revealed that empagliflozin preserved cardiac microvascular barrier function and integrity, sustained eNOS phosphorylation and endothelium-dependent relaxation, and increased microvessel density with amelioration of cardiac microvascular perfusion and of myocardial structure and function. Notably, the study suggested that all these effects of empagliflozin were generated through the inhibition of mitochondrial fission in an AMPK-dependent manner. More specifically, empagliflozin restored the AMP-to-ATP ratio to trigger AMPK activation, reduced the phosphorylation of Dynamin-related protein 1 (Drp1) at Ser616, and increased the Drp1 phosphorylation at Ser637, ultimately inhibiting the activation of Drp1, the critical effector of mitochondrial fission. In turn, the inhibition of mitochondrial fission preserved CMEC barrier function and viability by suppressing mitochondrial ROS production and oxidative stress. The empagliflozin-induced inhibition of mitochondrial fission also promoted CMEC migration through the amelioration of F-actin depolymerization and the formation of new micro-vessels [283].

Based on these results and on the role of mitochondrial damage in cardiac micro-vascular I/R injury, Cai et al. investigated whether empagliflozin could protect against this condition by sustaining mitochondrial homeostasis [284,285]. The study conducted both in vivo in mice subjected either myocardial I/R injury or a sham operation, and in vitro in CMECs isolated from mice after myocardial I/R injury, demonstrated that a seven-day in vivo pre-treatment with empagliflozin augmented FUNDC1-dependent mitophagy through AMPKα1 activation and ULK1 phosphorylation. The activation of mitophagy normalized mitochondrial fission/fusion, reduced endothelial oxidative stress, and hampered mitochondrial apoptotic signaling, thereby preserving EC homeostasis and microvessel integrity during microvascular I/R injury. Consistently, empagliflozin activated mitochondrial fission and suppressed mitochondrial fusion in I/R-treated CMECs. Since genetic ablation of FUNDC1 or AMPKα1 prevented empagliflozin from inhibiting mitochondrial oxidative stress, the study demonstrated that SGLT2 inhibition induced the AMPKa1/ULK1/FUNDC1 pathway, thus restoring mitophagy. This process, which is known to be largely suppressed at the myocardial reperfusion phase due to multiple mechanisms, represents important repair machinery for damaged and dysfunctional mitochondria through the normalization of mitochondrial fission/fusion [286,287,288,289]. The findings reported by Cai et al. likely elucidated the pathways by which SGLT2 inhibition provides a cardioprotective effect against I/R injury and could justify the infarct-sparing effect observed in hearts from diabetic and nondiabetic rats treated for 4 weeks with canagliflozin [285,290]. In other studies investigating the heart under hypoxia and reoxygenation conditions, empagliflozin increased ATP production through the AMPK/ACC pathway and attenuated mitochondrial O^−2^ generation by triggering the cardiac LKB1/AMPK signaling pathway [291], whereas dapagliflozin was shown to restore autophagic flux and to inhibit inflammation by promoting a selective autophagy degradation of the inflammasome component NLRP3 [292].

Other researchers have specifically investigated the impact of SGLT2-Is on the pathophysiology of HFpEF. As suggested by the study of Juni et al., a possible mediation of empagliflozin cardioprotective effects by NO transduction between ECs and non-ECs was implicated in an animal model of heart failure [274,293]. The investigators administered empagliflozin to mice suffering from LV pressure overload generated by transverse aortic constriction, a condition associated with the heart reduction of vascular endothelial growth factor-A, capillary rarefaction, and tissue hypoxia, and the progression of systolic dysfunction [294,295]. Overall, as shown in in vivo evaluation, in vitro experiments, and histological examinations, empagliflozin significantly improved the capillary rarefaction and endothelial apoptosis observed in hearts subjected to LV pressure, through the activation of the AKT/eNOS/NO pathway in ECs. In more detail, empagliflozin increased citrulline levels and decreased arginine content in cardiac tissue, consistent with an enhanced metabolism from arginine to citrulline and NO, through phosphorylation of eNOS in cardiac ECs. On the contrary, the eNOS inhibition attenuated the cardioprotection exerted by gliflozin.

Kolijn et al. explored the acute effects induced by in vivo treatment with empagliflozin in myocardium from patients with HFpEF and from obese ZDF rats as a model of HFpEF, with the aim to identify the mechanisms contributing to favorable clinical outcomes demonstrated by RCTs [296]. The authors found that empagliflozin significantly suppressed the increased levels of ICAM-1, VCAM-1, TNF-a, and IL-6, and attenuated the cytosolic and mitochondrial pathological oxidative parameters (H_2_O_2_, 3-nitrotyrosine, GSH, and lipid peroxide) characteristic of HFpEF. By reducing the myocardial inflammation and oxidative stress, empagliflozin improved endothelial function, thereby reversing the pathological repression of the NO–sGC–cGMP–PKG pathway and its downstream targets. The increased activity of PKG1α enhanced myofilament phosphorylation and reduced cardiomyocyte passive stiffness, with consequent correction of diastolic dysfunction.

In a further investigation, Cappetta et al. addressed the effects of dapagliflozin in salt-sensitive Dahl rats, a non-diabetic model of progressive hypertension, metabolic alterations, and cardiac and renal disturbances, as commonly observed in HFpEF patients [297]. Several features of coronary endothelial activation and dysfunction were confirmed in this model, such as the upregulation of VCAM-1 and E-selectin and the downregulation of eNOS. Echo-Doppler and heart catheterization documented an amelioration of diastolic function by dapagliflozin, although no direct action on isolated cardiomyocytes was observed. Rather, dapagliflozin was able to reverse eNOS deficit and to reduce cardiac inflammation and pro-fibrotic signaling. The potential involvement of the coronary endothelium was supported by the endothelial upregulation of Na^+^/H^+^ exchanger 1 in vivo, and by the direct effects of dapagliflozin on the activity of this exchanger in HUVECs in vitro. The authors hypothesized that the observed positive cardiac effects could be, at least in part, due to the action of dapagliflozin on the coronary endothelium, which resulted in a lower degree of endothelial inflammation/dysfunction and fibrosis and, finally, in the amelioration of cardiac function [297].

In all the in vitro findings we have described the anti-inflammatory and antioxidative effects of SGLT2-Is were demonstrated on resting endothelium, whereas ECs in vivo are constantly exposed to mechanical forces such as cyclic stretch. A study using HCAECs indicated that these drugs may protect from barrier dysfunction and increased permeability determined by cyclic stretch, an effect most likely mediated through the inhibition of NO scavenging by ROS and the attenuation of oxidative stress [298].

#### 5.2.2. Clinical Studies

The effect of empagliflozin on CFR or similar parameters was explored in a series of small RCTs, all of which included few participants and did not previously ascertain the presence of CAD.

Modest hemodynamic changes evaluated using 15O-H2O PET/CT were found in thirteen T2DM individuals studied before and after 4 weeks of treatment with empagliflozin or placebo [299]. In detail, empagliflozin reduced resting MBF by 13% (p < 0.01), but did not significantly affect adenosine stress MBF or MFR.

In another trial examining 90 patients with T2DM and known cardiovascular disease or high CV risk, no change to MFR, measured by cardiac 82Rb-PET/CT, was apparent at week 13 of empagliflozin treatment in either the drug or placebo group [300]. The authors hypothesized that the lack of effect could result from the only moderately reduced mean MFR at a baseline of 2.21, with many participants falling within the normal range, compared to previously reported median values as low as 1.6 in diabetic patients [19].

In line with this negative finding, a recent trial found that after treatment with empagliflozin for 12 weeks, in 19 of the 33 enrolled T2DM patients who completed the study, there was no improvement in CFVR measured by TTDE and adenosine stress [301]. However, since the study measured the non-endothelial dependent coronary vascular function, a possible effect of empagliflozin on endothelial function of the coronary microvessels could not be ruled out. Moreover, all patients had normal LVEF and a baseline CFVR of 2.60, a value above the cut-off level for CMD used in prognostic studies [302]. Once again, the small population size of patients with normal HF and little alteration of baseline CFVR could be responsible for negative results, considering that both EMPAREG and DAPA-HF studies documented a most benefit from SGLT2-I treatment in patients with HF [251,303].

Greater improvement to vascular markers and effective cardiac function was shown in 160 T2DM patients after twelve months of an add-on treatment of metformin with GLP-1RA, SGLT2 Is, and their combination with respect to insulin [304]. In addition, at baseline and at 4 and 12 months of treatment, measurements of the perfused boundary region (PBR) of the sublingual arterial microvessels, a marker of endothelial glycocalyx thickness, were made. After twelve months, patients under the use of both GLP-1RAs and SGLT2-Is had a remarkable increase in endothelial glycocalyx thickness, as assessed by PBR. The glycocalyx is a gel-like layer of proteoglycans, glycoproteins, and adsorbed plasma proteins, lining the luminal surface of the endothelium which functions as a fluid mediating shear-induced release of NO by endothelial cells and as barrier that protects vessel walls from the circulating inflammatory cells [305]. The results obtained from sublingual microvessels can theoretically be translated to the coronary microcirculation and thereby contribute to the improvement of cardiac function.

**Table 2:** Effects of SGLT2-Is on the coronary microvascular compartment.

Preclinical Studies					
Experimental Model	Treatment	Duration of Treatment	Effects	Suggested Mediating Mechanisms	Ref.
co-culture model of human CMECs and adult rat cardiomyocytes stimulated by TNF-α	empagliflozin		correction of TNF-α-mediated impairment of CMEC cardiomyocyte interaction	reduction of mitochondrial ROS level, prevention of cytoplasmic ROS buildup and restoration of NO bioavailability	[274]
co-culture model of human CMECs and adult rat cardiomyocytes exposed at uremic serum	empagliflozin		restoration of NO level in ECs	reversion of mitochondrial fission, responsible of ↑ ROS production and intracellular accumulation	[279]
mice with metabolic syndrome and pre/early diabetes	empagliflozin	10 weeks	↑ CFVR, no difference in CD31 staining, and ↑ L-arginine and ↑ L-arginine/ADMA	NO-dependent improvement of endothelial function	[269]
STZ-induced diabetic mice	empagliflozin or an inhibitor of mitochondrial fission	20 weeks	preserved cardiac microvascular barrier, sustained eNOS phosphorylation and endothelium-dependent relaxation, and increased microvessel density	↓ mitochondrial fission through ↓ activation of Drp1	[283]
mice subjected to myocardial I/R injury and CMECs from mice after myocardial I/R injury	in vivo pre-treatment with empagliflozin	7 days	normalized mitochondrial fission/fusion, ↓ endothelial oxidative stress and hampered mitochondrial apoptotic signaling	↑ FUNDC1-dependent mitophagy through AMPKα1 activation and ULK1 phosphorylation	[285]
mice model of HF (transv. aortic constriction)	empagliflozin	2 weeks	improved capillary rarefaction and endothelial apoptosis	↑ AKT/eNOS/NO pathway in ECs	[293]
yocardium from a rat model of HFpEF	empagliflozin	in vivo acute treatment	improved endothelial function by reduced oxidative stress and inflammation	reversed repression of NO–sGC–cGMP–PKG pathway and its downstream targets	[296]
Dahl salt-sensitive rats as a model of HFpEF	dapagliflozin	6 weeks	↓ endothelial inflammation/dysfunction		[297]
**Clinical Studies**					
**Subjects**	**Treatment**	**Duration of Treatment**	**Effects**	**Diagnostic Modality**	**Ref.**
13 T2DM people or placebo	empagliflozin	4 weeks	↓ MBF by 13% and no variation of adenosine stress MBF or MFR	^15^O-H_2_O PET/CT	[299]
90 T2DM patients with known CVD or high CV risk	empagliflozin or placebo	13 weeks	no change in MFR	^82^Rb-PET/CT	[300]
19 T2DM patients	empagliflozin	12 weeks	no improvement in CFVR	TTDE with adenosine stress	[301]
160 T2DM patients	add-on to metformin of GLP-1RA, SGLT2 Is, or both, vs. insulin	12 months	increase of endothelial glycocalyx thickness	measurements of perfused boundary region of sublingual arterial microvessels	[304]

## 6. Conclusions

Dysfunction of the coronary microcirculation, a tightly regulated system meant to match myocardial perfusion to metabolic demands, is a hallmark of diabetes-induced microvascular damage. Due to hyperglycemia and insulin resistance, diabetes affects both the injury and repair processes of microvascular compartments with peculiar mechanisms with respect to other vascular diseases. In particular, the increase of metabolites associated with hyperglycemia in microvessel cells can cause specific functional and structural changes mediated by PKC or ROS activation and exacerbated by the accumulation of non-diabetes-specific toxic substances, such as oxidants, AGE, and methylglyoxal.

Failure of normally functioning coronary microvasculature and the associated remodeling that occurs over time as a compensatory method of restoring normal microvascular wall tension, represent early sub-clinical culprits of heart disease and cardiac mortality in diabetic patients. Therefore, CMD could represent an important therapeutic target in preventing, delaying, or reversing these complications.

Unfortunately, our present therapeutic armamentarium for the treatment of CMD is very limited. In this context, multiple encouraging findings have resulted from preclinical studies on the beneficial effects exerted by GLP-1-based and SGLT2 inhibition therapy, even if they are not convincingly confirmed by the few small clinical trials that we have available to date.

In the coming years, this will likely result, in a wide range of these therapies in the diabetic population, which will allow for analysis through clinical studies of the real-life eventual benefits related to CMD. Meanwhile, an extension of experimental investigations is advisable in order to reinforce the promise of these drugs for the prevention and treatment of CMD. Moreover, further research into the pathophysiologic aspects that we do not yet understand, including genetics, proteomics, metabolomics, and others, is of capital importance in order to unveil other targeted therapeutic approaches for this harmful complication, including outside of diabetes.

## Data Availability

Not applicable.

## Abbreviations

ACh: acetylcholine; AGEs: advanced glycation end products; AMPK: AMP-activated protein kinase; Ang II: angiotensin II; BH4: tetrahydrobiopterin; BMI: body mass index; CAD: coronary artery disease; CBF: coronary blood flow; CFR: coronary blood reserve; CFVR: coronary flow velocity ratio; CKD: chronic kidney disease; CMD: coronary microvascular dysfunction; CMECs: coronary microvascular endothelial cells; CMR: coronary magnetic resonance; CT: computed tomography; CV: cardiovascular; CVD: cardiovascular disease; DAG: diacylglycerol; DM: diabetes mellitus; ECs: endothelial cells; EDHF: endothelium-deriving hyperpolarizing factor; EF: ejection fraction; eNOS: endothelial nitric oxide synthase; ET-1: endothelin-1; FDA: food and drug administration; GLP-1: Glucagon like peptide 1; GLP-1 RAs: glucagon like peptide 1 receptor agonists; GSH: reduced glutathione; GTP: guanosine triphosphate; HAECs: human amniotic epithelial cells; HbA1c: glycosylated hemoglobin; HCAECs: human coronary artery endothelial cells; HDL: high density lipoprotein; HF: heart failure; HFpEF: heart failure with preserved ejection fraction; HFrEF: heart failure with reduced ejection fraction; HG: high glucose; H_2_O_2_: hydrogen peroxide; HUVECs: human umbilical vein endothelial cells; ICAM-1: intercellular adhesion molecule-1; IL: interleukins; INOCA: ischemia and non obstructive coronary arteries; I/R: ischemia/reperfusion injury; LDL: low density lipoprotein; LV: left ventricular; MBF: myocardial blood flow; MFR: myocardial flow reserve; MI: myocardial infarction; MINOCA: myocardial infarction and non obstructive coronary arteries; MPR: myocardial perfusion reserve; MPRI: myocardial perfusion reserve index; NF-κB: nuclear factor-κB; NO: nitric oxide; NOX: NADPH oxidase; O^−2^: superoxide anion; PCI: percutaneous coronary intervention; PDGF: platelet-derived growth factor; PET: positron emission tomography; PKC: protein kinase C; RAGE: receptor for advanced glycation end products; RCTs: randomized clinical trials; ROS: reactive oxygen species; SGLT2: sodium-glucose cotransporter-2; SOD: superoxide dismutase; STZ: streptozotocin; T1DM: type 1 diabetes mellitus; T2DM: type 2 diabetes mellitus; TGF-β: transforming growth factor-β; TNF-α: tumor necrosis factor-α; TTDE: transthoracic doppler echocardiography; VCAM-1: vascular cell adhesion molecule 1; VSMCs: vascular smooth muscle cells; XO: xantine oxidase.
